# Burden of Intrapancreatic Fat Deposition in Type 2 Diabetes and the Role of Obesity: A Systematic Review and Meta-Analysis

**DOI:** 10.1007/s13679-026-00718-3

**Published:** 2026-05-21

**Authors:** Hyun Hee Sul, Saeed Ahmad, Pojsakorn Danpanichkul, Alessandro Mantovani, Matheus Souza, Maxim S. Petrov

**Affiliations:** 1 Department of Internal Medicine, Jersey City Medical Center/Rutgers Health, Jersey City, NJ USA; 2 Beth Israel Deaconess Medical Center, Harvard Medical School, Boston, MA USA; 3 Department of Internal Medicine, Texas Tech University Health Sciences Center, Lubbock, TX USA; 4 Department of Medicine, University of Verona, Verona, Italy; 5 Metabolic Disease Research Unit, IRCCS Sacro Cuore - Don Calabria Hospital, Negrar di Valpolicella, VR Italy; 6 Department of Internal Medicine, Federal University of Rio de Janeiro, Rio de Janeiro, RJ Brazil; 7 School of Medicine, University of Auckland, Auckland, New Zealand

**Keywords:** Intrapancreatic fat deposition, Metabolism, Pancreas, Diabetes mellitus, MRI

## Abstract

**Background:**

Growing evidence supports a role for high intrapancreatic fat deposition (IPFD) in the pathogenesis of type 2 diabetes mellitus (T2DM); however, the magnitude of this association and the extent to which it is influenced by body mass index (BMI), liver fat content (LFC), and age remain uncertain.

**Objective:**

To quantitatively evaluate IPFD measured by magnetic resonance imaging (MRI) in individuals with T2DM and to investigate study-level factors contributing to between-study variability.

**Methods:**

We systematically searched PubMed and Embase for observational studies comparing MRI-measured IPFD in individuals with T2DM versus non-diabetic controls, excluding studies in which IPFD quantification was performed using AI-based models. Pooled standardized mean differences (SMDs) were estimated using a restricted maximum likelihood approach with Hartung-Knapp adjustment. Heterogeneity was explored through subgroup, meta-regression, and sensitivity analyses.

**Results:**

Thirty studies (*n*=3,980) were included. Individuals with T2DM had significantly higher IPFD than non-diabetic controls (SMD 1.13, 95% CI 0.74 to 1.51), with no significant differences when stratified by pancreatic region, MRI technique, geographic region, sample size, and risk of bias. In multivariable models, BMI remained the only consistent moderating covariate, and the excess IPFD in T2DM persisted after adjusting for BMI, LFC, and age (intercept=0.797, p=0.010, false discovery rate-adjusted p=0.018). Advanced heterogeneity exploration identified no structural outliers, and the pooled effect size remained consistent across multiple sensitivity analyses.

**Conclusion:**

T2DM is associated with substantially higher MRI-quantified IPFD, independent of study-level differences in BMI, LFC, and age. Although causality cannot be inferred, these findings support the hypothesis that IPFD may be involved in the underlying mechanism of T2DM. They also suggest that high IPFD could represent a metabolic hallmark, with potential implications for risk stratification and therapeutic targeting.

**Graphical Abstract:**

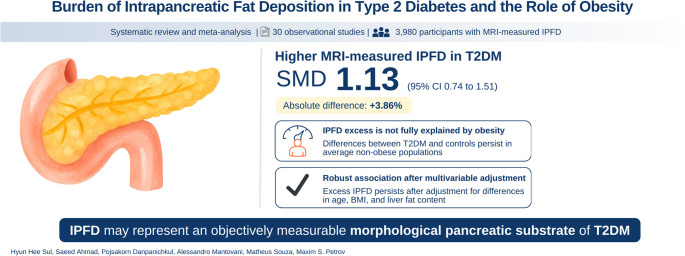

**Supplementary Information:**

The online version contains supplementary material available at 10.1007/s13679-026-00718-3.

## Introduction

Intrapancreatic fat deposition (IPFD) has emerged as an important component of ectopic fat accumulation and a potential contributor to systemic metabolic dysfunction, extending beyond the well-known sites of the liver and visceral adipose tissue [1]. Over the past decade, IPFD has attracted increasing scientific attention, driven largely by advances in imaging-based characterization of multi-organ ectopic fat burden [2]. While the pancreas normally contains a small amount of fat, excessive IPFD has been increasingly associated with β-cell dysfunction, insulin resistance, and alterations in the islet microenvironment [3–6]. Further, longitudinal cohort studies indicate that excessive IPFD is independently associated with a higher risk of incident type 2 diabetes mellitus (T2DM) [7–10]. 

A key challenge in studying IPFD is that its biological burden in T2DM remains largely unknown. Specifically, the extent to which IPFD increases during the natural course of T2DM, in the absence of interventions, has not been clearly determined. Although several meta-analyses have explored associations between IPFD and T2DM, they have primarily focused on risk ratios or odds ratios, which depend heavily on the imaging modality and the thresholds used to define excessive IPFD [3, 11–14]. Techniques such as transabdominal and endoscopic ultrasound are highly operator-dependent and qualitative, making them unreliable for establishing excessive IPFD. Computed tomography estimates IPFD only indirectly using X-ray attenuation values [15, 16], whereas magnetic resonance spectroscopy (MRS) quantifies IPFD directly but is susceptible to voxel placement errors and partial volume effects, including contamination from surrounding visceral fat [1, 5, 17]. As such, both techniques remain suboptimal for accurate IPFD measurement. In contrast, chemical-shift-encoded magnetic resonance imaging (MRI), which enables quantitative assessment of proton density fat fraction (PDFF), is a well validated and widely used non-invasive method for the quantification of IPFD [18]. It permits evaluation of the extent to which IPFD differs between individuals with T2DM and non-diabetic controls. For understanding the role of IPFD in T2DM, the magnitude of such differences provides more meaningful insight than prevalence above arbitrary thresholds. These quantitative insights could also guide the development and targeting of interventions, with implications for improved care of individuals with T2DM [19–21]. Another important factor that remains underexplored in the relationship between IPFD and T2DM is obesity. Although body mass index (BMI) is often assumed to correlate directly with IPFD, no systematic studies have rigorously assessed the role of obesity in modulating this association. Other factors potentially influencing the relationship between IPFD and T2DM have also not been systematically investigated.

The primary aim of this study was to conduct a systematic literature review and meta-analysis to quantify the excess IPFD in individuals with T2DM compared with non-diabetic controls. The secondary aim was to examine the roles of obesity and other potential contributing factors in this relationship.

## Methods

### Data Sources and Searches

This systematic review followed the Preferred Reporting Items for Systematic reviews and Meta-Analyses (PRISMA) and Meta-analysis Of Observational Studies in Epidemiology (MOOSE) guidelines [22, 23]. The review protocol was prospectively registered in the International Prospective Register of Systematic Reviews (PROSPERO) database (CRD420251208380). We systematically searched two major electronic databases (PubMed and Embase) from inception till November 15, 2025. The predefined search strategy is detailed in the Supplementary Methods. In addition, reference lists of included studies and relevant reviews were manually screened, and the authors’ personal libraries were consulted to identify any additional eligible studies.

### Eligibility Criteria

After deduplication, two authors (H.H.S. and S.A.) independently screened study titles and abstracts. Full-text articles were then assessed for eligibility according to the predefined inclusion criteria. Any discrepancies were resolved by consultation with a third author (M.S.). Studies were eligible for inclusion if they met the following criteria: (i) observational design and inclusion of patients diagnosed with T2DM; (ii) quantitative assessment of IPFD using chemical shift-encoded MRI (e.g., Dixon, PDFF); (iii) provision of sufficient data to calculate differences in IPFD between patients with T2DM and non-diabetic control participants. The diagnosis of T2DM was accepted as reported in the primary studies. Control participants were defined as individuals without diabetes or prediabetes. Interventional studies (randomized or non-randomized) were excluded, as these predominantly assess changes in IPFD rather than baseline burden and may therefore confound evaluation of the natural IPFD burden [19–21, 24, 25]. Studies using AI–based methods for quantification of IPFD were excluded, as these modern approaches currently demonstrate suboptimal accuracy compared with manual quantification [26]. Studies that quantified IPFD using H^1^-MRS were also excluded [17]. Only original articles were considered for inclusion. Studies that were conference abstracts, focused on highly selected populations (e.g., pediatric, gestational diabetes mellitus, individuals after acute pancreatitis), non-original works (e.g., guidelines, editorials, and reviews), or had a small total sample size (< 20 participants) were excluded. When multiple reports from the same cohort were identified, only the most comprehensive publication was included to avoid data duplication.

### Data Extraction and Risk of Bias Assessment

The following study-level data were extracted using a standardized form: (i) study characteristics: first author, publication year, location, study year, setting/population, MRI characteristics, and T2DM diagnosis; (ii) population characteristics: sample size, age, sex, BMI, liver fat content [LFC], HbA1c levels; and (iii) outcome of interest. Data transformations were performed according to the guidelines in the Cochrane Handbook and the recommended formulas, when applicable [27]. Consistent with prior MRI studies, whole-pancreas IPFD was estimated using the mean of the three pancreatic regions of interest (ROIs: head, body, and tail) when volume-weighted measures were unavailable [28]. If studies reported IPFD measures for only a single ROI, these data were used exclusively in the corresponding subgroup analysis [29]. When data were only available as graphs or bar charts [2], values were manually extracted using a free web-based digitizing tool (WebPlotDigitizer v4.2), following the recommended methods for standardized quantitative synthesis [30]. Two authors (S.A. and H.H.S.) independently assessed the risk of bias in each study using the Newcastle-Ottawa Scale [31]. Disagreements were resolved through evaluation by a third author (M.S.). Publication bias was evaluated using visual inspection of funnel plot asymmetry.

### Outcomes of Interest

The primary outcome was the standardized mean difference (SMD) in IPFD between patients with T2DM and non-diabetic controls, as quantified by chemical shift-encoded MRI. To improve the clinical interpretability of IPFD differences, we also estimated mean differences (MDs) in the overall analysis.

### Data Synthesis and Statistical Analysis

All statistical analyses were performed using R software (v4.4.0; “meta” and “metafor” packages). A two-tailed p-value < 0.05 was considered statistically significant. Pooled SMDs/MDs and their 95% confidence intervals (CIs) were estimated using the restricted maximum likelihood (REML) method and the Hartung-Knapp adjustment to provide robust variance estimates [32]. Heterogeneity was evaluated using the between-study variance (τ^2^) and the I² statistic. To investigate potential sources of heterogeneity, subgroup analyses were performed according to specific pancreatic region assessed, MRI-based technique, world location, sample size, and risk of bias. We then performed univariable meta-regressions, using study-level SMDs for age, BMI and LFC, as well as proportion of women as potential moderators of the associations with SMD IPFD. Given the expected interdependence between IPFD and metabolic traits, we further performed multivariable meta-regressions using prespecified models (Model 1: SMD BMI + SMD Age; Model 2: SMD BMI + SMD LFC; Model 3: SMD BMI + SMD Age + SMD LFC). Importantly, women proportion was not included in the multivariable models because, unlike the SMD-based moderators, it reflected study composition rather than a between-group contrast. The proportion of heterogeneity explained by each meta-regression model was summarized using the R^2^ analog. To account for multiple testing, p-values for moderator coefficients were adjusted using the Benjamini-Hochberg false discovery rate (FDR) procedure [33]. Because the intercept of the most adjusted model was also used to assess whether excess IPFD persisted after covariate adjustment, we additionally applied FDR correction including the model intercepts. FDR-adjusted p-values (i.e., q-values) < 0.05 were considered statistically significant. To characterize the structure of heterogeneity, we conducted Graphic Display of Study Heterogeneity (GOSH) analyses using 50,000 random subsets of studies under the REML estimator [34]. Density-based clustering (DBSCAN) was applied to the GOSH space (pooled SMD × τ^2^) to identify homogeneous clusters and potential outlier substructures. We conducted a sensitivity analysis restricting the analysis to studies with an average non-obese population. Obesity status was determined using the standard World Health Organization cutoff for non-Asian populations (BMI ≥ 30 kg/m²) and an adjusted cutoff for Asian populations (BMI ≥ 28 kg/m²). Leave-one-out sensitivity analysis was also performed to evaluate whether any single study disproportionately affected the pooled SMD. Post hoc analysis was performed by restricting the meta-analysis to the studies within the DBSCAN-identified homogeneous cluster.

## Results

### Search Results

**Fig. 1:**
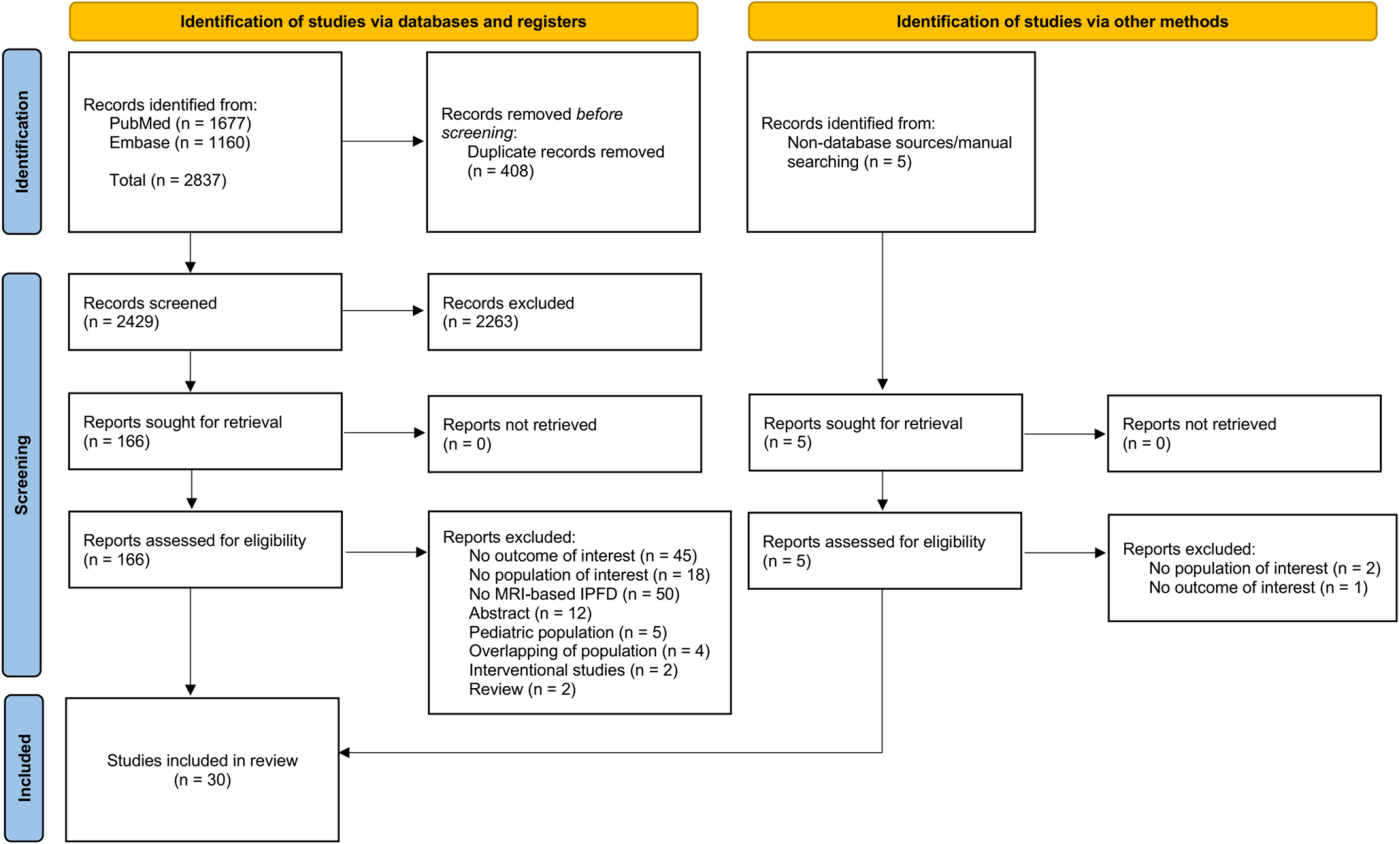
PRISMA flow diagram. PRISMA, Preferred Reporting Items for Systematic reviews and Meta-Analyses

A comprehensive literature search yielded 2,837 records, of which 408 duplicates were removed. After title and abstract screening, 2,429 records were assessed and 166 studies were retrieved for full-text eligibility evaluation. We also identified 2 additional eligible studies via non-database sources and manual searching [35, 36]. Ultimately, 30 studies (*n* = 3,980 individuals) were included in the meta-analysis [2, 28, 29, 35–61]. Fig. 1 presents the PRISMA diagram summarizing the study selection processes.

## Characteristics of Included Studies

The main characteristics of the included studies are presented in Table 1. Most studies were cross-sectional in design (90%) [28, 29, 36–56, 58–61] and conducted in clinic or hospital-based settings (90%) [2, 28, 29, 35, 36, 38–46, 48–54, 56–61] . Geographically, the majority were performed in Asia (60%) [29, 36, 40–42, 45, 46, 48–54, 56, 58, 60, 61] . MRI characteristics are detailed in Table S1. Most studies reported whole-pancreas IPFD measurement (86.7%) [2, 35–57, 59, 60], with a subset also providing region-specific measurements [2, 40, 42, 44, 46, 49, 56]. Three studies reported only region-specific values for the head, body, and tail [28, 58, 61], whereas one study reported data exclusively for the pancreatic head [29]. Baseline patient characteristics stratified by T2DM status are reported in Table S2. Twelve studies were judged to have a low risk of bias based on the Newcastle–Ottawa Scale [2, 28, 43, 47, 49–51, 54, 58–61], and Table S3 summarizes the risk of bias assessments for all included studies.

**Table 1:** Main characteristics of the included studies

First author (year)	Country	Study design	MRI technique	Pancreatic location	Sample size, *n*^#^	T2DM, %
Ma et al. [29] (2014)	China	Cross-sectional	Multi-echo Dixon (6-echo)	Regions only (head)	34	70.6
Kühn et al. [37] (2015)	Germany	Cross-sectional	Three-echo Dixon	Whole pancreas only	810	8.6
Idilman et al. [38] (2015)	Turkey	Cross-sectional	Multi-echo Dixon (6-echo)	Whole pancreas only	41	12.2
Macauley et al. [39] (2015)	UK	Cross-sectional	Three-echo Dixon	Whole pancreas only	55	74.5
Chai et al. [40](2016)	China	Cross-sectional	Two-echo Dixon	Whole pancreas and regions (head, body, tail)	100	70.0
Heber et al. [2] (2017)	Germany	Case-control	Multi-echo Dixon (6-echo)	Whole pancreas and regions (head, body, tail)	290	18.3
Lu et al. [41] (2019)	China	Cross-sectional	Three-echo Dixon	Whole pancreas only	113	69.0
Wang et al. [42] (2019)	China	Cross-sectional	Multi-echo Dixon (6-echo)	Whole pancreas and regions (head, body, tail)	31	48.4
Tirkes et al. [43] (2019)	USA	Cross-sectional	Two-echo Dixon	Whole pancreas only	118	13.6
Nadarajah et al. [28] (2020)	USA	Cross-sectional	Two-echo Dixon	Regions only (head/body/tail)	195	23.1
Sarma et al. [44] (2020)	USA	Cross-sectional	Multi-echo Dixon (6-echo)	Whole pancreas and regions (head, body/tail)	27	51.9
Li et al. [45] (2021)	China	Cross-sectional	Three-echo Dixon	Whole pancreas only	60	11.7
Zheng et al. [46] (2022)	China	Cross-sectional	Two-echo Dixon	Whole pancreas and regions (head, body, tail)	157	75.2
Waddell et al. [47] (2022)	UK	Cross-sectional	Multi-echo Dixon (10-echo)	Whole pancreas only	266	49.2
Wen et al. [48] (2022)	China	Cross-sectional	Multi-echo Dixon (6-echo)	Whole pancreas only	34	47.1
Yu et al. [49] (2023)	China	Cross-sectional	Multi-echo Dixon (6-echo)	Whole pancreas and regions (head, body, tail)	82	34.1
Yi et al. [50] (2023)	China	Cross-sectional	Multi-echo Dixon (6-echo)	Whole pancreas only	95	49.5
Cao et al. [51] (2023)	China	Cross-sectional	Multi-echo Dixon (6-echo)	Whole pancreas only	92	43.5
Yasokawa et al. [52] (2023)	Japan	Cross-sectional	Multi-echo Dixon (6-echo)	Whole pancreas only	55	41.8
Ting et al. [53] (2023)	China	Cross-sectional	Three-echo Dixon	Whole pancreas only	100	70.0
Wang et al. [36] (2023)	China	Cross-sectional	Two-echo Dixon	Whole pancreas only	100	80.0
An et al. [54] (2024)	China	Cross-sectional	Multi-echo Dixon*	Whole pancreas only	341	19.9
Diamond et al. [55] (2024)	UK	Cross-sectional	Multi-echo Dixon*	Whole pancreas only	203	55.2
Qu et al. [56] (2025)	China	Cross-sectional	Multi-echo Dixon*	Whole pancreas and regions (head, body, tail)	80	50.0
Elsayed et al. [57] (2025)	Egypt	Case-control	Multi-echo Dixon (6-echo)	Whole pancreas only	30	50.0
Yuan et al. [58] (2025)	China	Cross-sectional	Multi-echo Dixon (6-echo)	Regions only (head/body/tail)	118	67.8
Akhan et al. [59] (2025)	Turkey	Cross-sectional	Two-echo Dixon	Whole pancreas only	86	64.0
Nie et al. [60] (2025)	China	Cross-sectional	Multi-echo Dixon*	Whole pancreas only	96	47.9
Nielsen et al. [35] (2025)	Denmark	Case-control	Multi-echo Dixon*	Whole pancreas only	23	52.2
Zhu et al. [61] (2025)	China	Cross-sectional	Multi-echo Dixon*	Regions only (head/body/tail)	148	50.0

* In these instances, the exact number of echoes was not clearly reported

^**#**^ In studies where a subset of participants with prediabetes was identified, these individuals were excluded from the analysis

*Abbreviations*: MRI magnetic resonance imaging, T2DM type 2 diabetes mellitus

### Burden of Intrapancreatic Fat Deposition

**Fig. 2:**
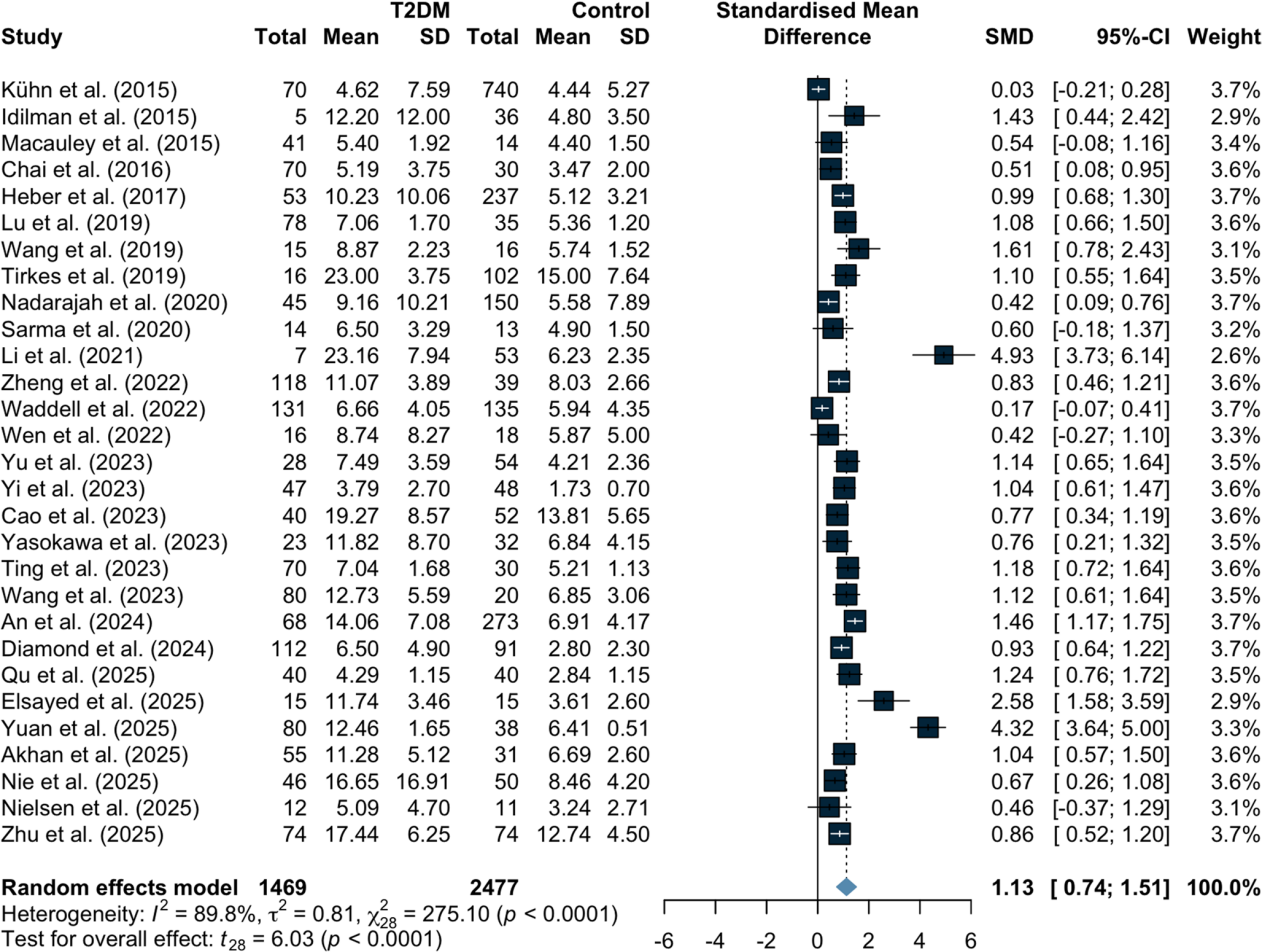
Differences in IPFD between type 2 diabetes and controls across all included studies. IPFD, intrapancreatic fat deposition

**Fig. 3:**
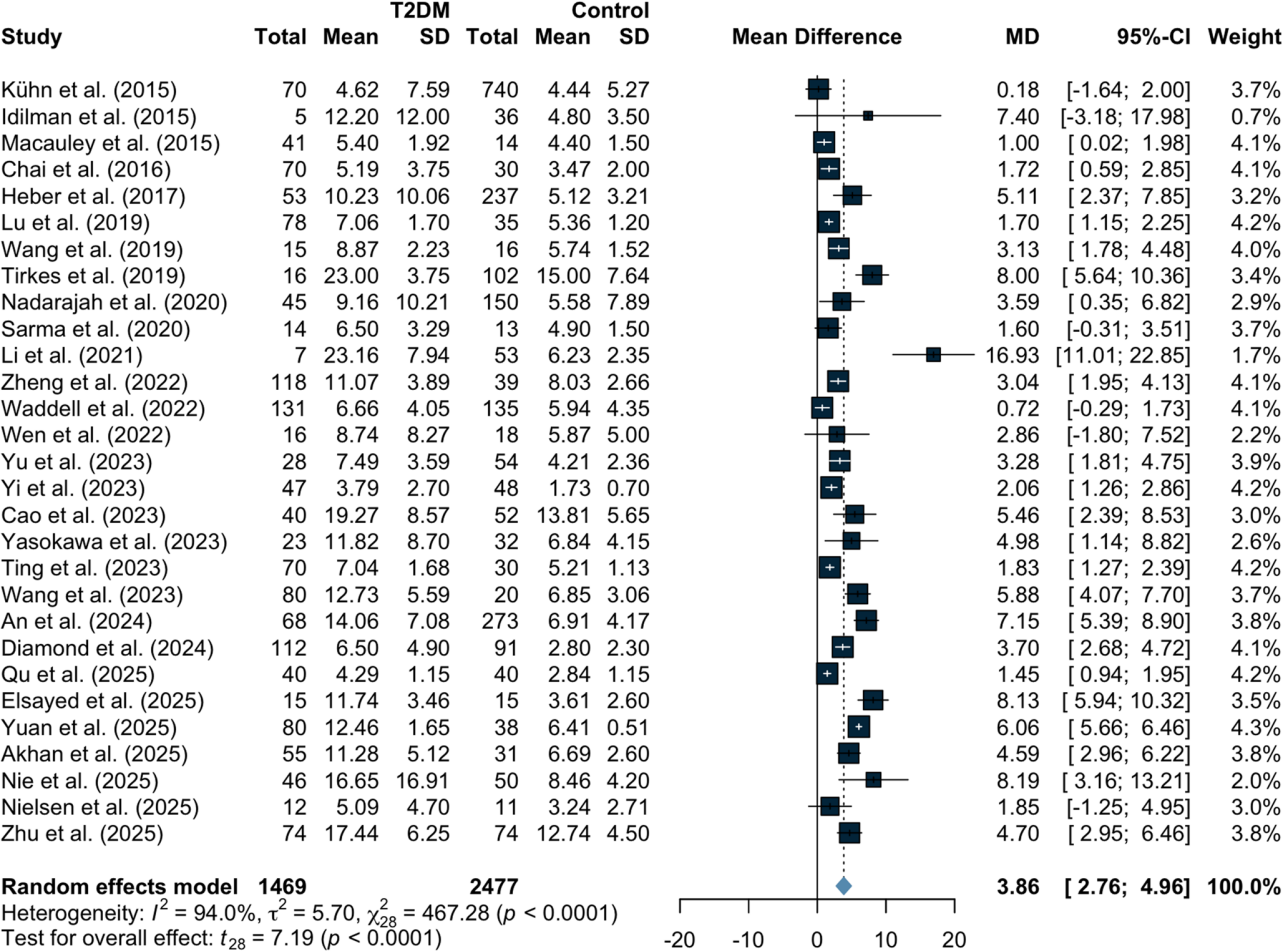
Absolute differences in IPFD between type 2 diabetes and controls across all included studies. IPFD, intrapancreatic fat deposition

Figure 2 shows the distribution of effect estimates across observational studies evaluating MRI-based IPFD in participants with T2DM. Overall, individuals with T2DM had significantly higher IPFD compared with non-diabetic controls (SMD 1.13, 95% CI 0.74 to 1.51; *p* < 0.0001), with substantial between-study heterogeneity (I²=89.8%, τ^2^ = 0.81). When expressed as absolute IPFD change, the pooled MD was 3.86% (95% CI 2.76 to 4.96, I²=94%, τ^2^ = 5.70; *p* < 0.0001; Fig. 3).

**Fig. 4:**
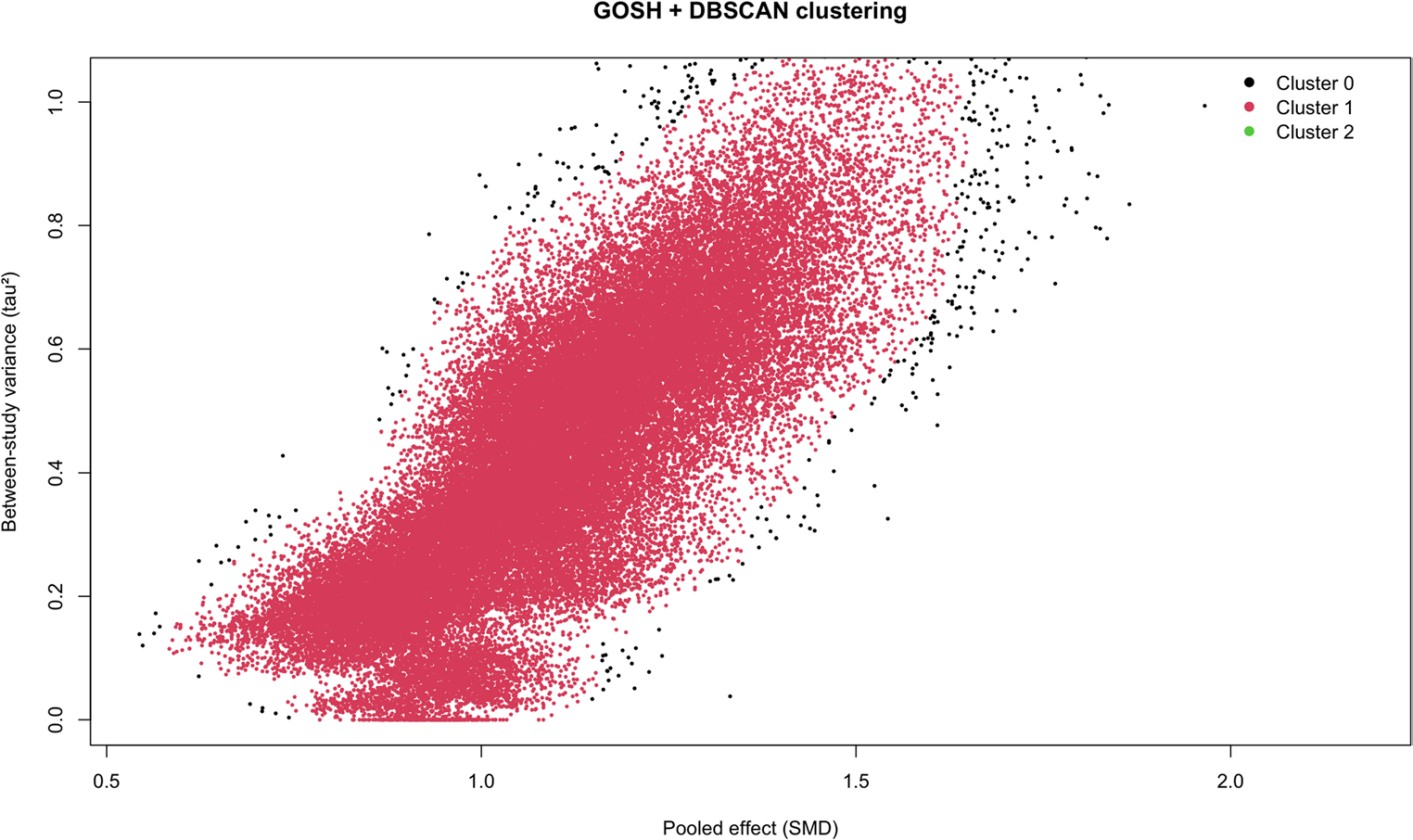
Advanced investigation of between-study heterogeneity. Graphic Display of Study Heterogeneity (GOSH) plot derived from 50,000 random subsets of the included studies, showing the relationship between pooled effect size (SMD) and between-study variance (τ^2^) estimated under random-effects modeling. Points are colored by DBSCAN cluster membership, which identified one dominant homogeneous core cluster (Cluster 1: 49,279/50,000 subsets) and a small proportion of subsets classified as noise (Cluster 0: 694/50,000), with a very small peripheral cluster (Clusters 2: 27/50,000)

GOSH exploration revealed a continuous heterogeneity landscape, with pooled SMDs spanning a wide range of τ^2^ values and no discrete “islands” suggestive of structural outliers (Fig. 4). Application of DBSCAN to the GOSH space identified one dominant cluster (cluster 1: 49,279/50,000 subsets; 98.6%), a small proportion of subsets classified as noise (cluster 0: 694/50,000; 1.4%), and very small peripheral clusters (clusters 2: 27/50,000; <0.1%). The dominant cluster exhibited the lowest mean τ² among non-noise clusters (mean τ² = 0.43) compared with the peripheral cluster (mean τ² = 1.16) and noise (mean τ² = 0.96), indicating a robust “core” region in the heterogeneity landscape. All studies were consistently represented within this main cluster (~ 49–51% across studies). Post hoc analysis restricted to studies within the dominant cluster yielded identical pooled estimates to the full model (SMD 1.13, 95% CI 0.75 to 1.51; I² = 94.6%, τ² = 0.83; *p* < 0.0001). A sensitivity analysis using the leave-one-out approach did not significantly affect the overall estimate (Figure S1).

### Influence of Obesity

**Fig. 5:**
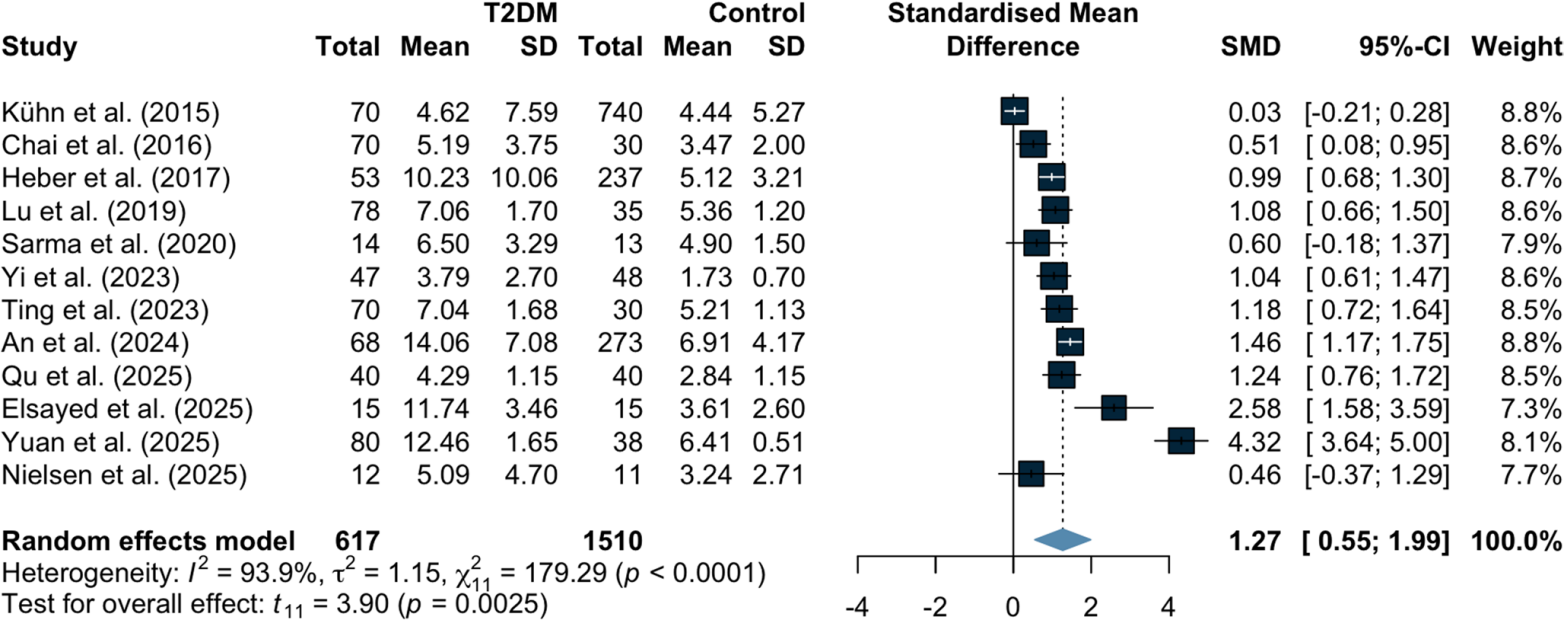
Differences in IPFD between individuals with type 2 diabetes and controls in average non-obese populations. IPFD, intrapancreatic fat deposition

A sensitivity analysis restricted to studies of average non-obese populations demonstrated that individuals with T2DM had significantly higher IPFD compared with controls (SMD 1.27, 95% CI 0.55 to 1.99; *p* = 0.0025; Fig. 5), with substantial between-study heterogeneity (I²=93.9%, τ^2^ = 1.15). In a univariable meta-regression, the SMD for BMI statistically significant (coefficient = 0.473, 95% CI 0.284 to 0.662; *p* < 0.0001) and explained 64.9% of the between-study heterogeneity. In multivariable meta-regressions, the SMD for BMI persisted statistically significant when the models included LFC (coefficient = 0.568, 95% CI 0.272 to 0.863; *p* = 0.001) and included LFC together with age (coefficient = 0.583, 95% CI 0.306 to 0.860; *p* = 0.0006). These models explained 79.89% and 80.44% of the between-study heterogeneity, respectively (Table 2). After FDR correction, SMD BMI coefficients remained statistically significant across all models. The proportion of women and SMD age were not statistically significant after correction, whereas SMD LFC was significant only in univariable analysis and did not remain significant in models including SMD BMI. When the FDR correction was extended to include model intercepts, the intercept of the most adjusted model remained significant (q = 0.018) (Table 2).

**Table 2:** Factors influencing differences in IPFD between individuals with type 2 diabetes and controls

	k	*R* ^2^		Estimate	95% confidence interval	*P*-value	Q-value*	Q-value^#^
**Univariable meta-regression**								
Women, %	22	3.29%	Intercept	1.911	0.556 to 3.266	0.008	-	0.017
			Women, %	−0.019	−0.048 to 0.010	0.191	0.318	0.274
SMD BMI	21	64.90%	Intercept	0.664	0.374 to 0.954	0.0001	-	< 0.001
			SMD BMI	0.473	0.284 to 0.662	< 0.0001	< 0.001	< 0.001
SMD Age	25	0%	Intercept	0.963	0.550 to 1.375	< 0.0001	-	< 0.001
			SMD Age	0.198	−0.461 to 0.856	0.541	0.624	0.592
SMD LFC	17	40.78%	Intercept	0.247	−0.494 to 0.988	0.489	-	0.591
			SMD LFC	1.041	0.328 to 1.754	0.007	0.018	0.017
**Multivariable meta-regression**								
SMD BMI and SMD Age	21	61.74%	Intercept	0.610	0.263 to 0.957	0.002	-	0.005
			SMD BMI	0.474	0.281 to 0.666	< 0.0001	< 0.001	< 0.001
			SMD Age	0.151	−0.343 to 0.645	0.529	0.624	0.592
SMD BMI and SMD LFC	16	79.89%	Intercept	0.873	0.279 to 1.468	0.007	-	0.017
			SMD BMI	0.568	0.272 to 0.863	0.0011	0.003	0.004
			SMD LFC	−0.199	−0.987 to 0.588	0.594	0.636	0.621
SMD BMI, SMD Age, and SMD LFC	16	80.44%	Intercept	0.797	0.234 to 1.360	0.010	-	0.018
			SMD BMI	0.583	0.306 to 0.860	0.0006	0.003	0.003
			SMD Age	0.395	−0.096 to 0.887	0.105	0.197	0.161
			SMD LFC	−0.271	−1.012 to 0.471	0.442	0.624	0.591

* FDR-adjusted p-values for moderator coefficients only

^#^ FDR-adjusted p-values from a broader correction including model intercepts

*Abbreviations*: R^2^: proportion of between-study heterogeneity explained by the moderator; SMD standardized mean difference, BMI body mass index, LFC liver fat content, FDR false discovery rate

### Influence of Other Factors

In subgroup analyses, stratification by region of the pancreas (*p* = 0.99), MRI technique (*p* = 0.85), world location (*p* = 0.08), sample size (*p* = 0.68), or risk of bias (*p* = 0.94) did not significantly affect the differences in IPFD between individuals with T2DM and non-diabetic controls (Table 3). The funnel plot was relatively asymmetric on visual inspection (Figure S2).

**Table 3:** Pooled IPFD differences across subgroups

Parameter	Studies, *n*	Patients, *n*	SMD	95% confidence interval	I², %	τ^2^	*P*-value
By region of the pancreas							
Head	11	1262	1.24	0.25 to 2.23	93.6	2.02	0.99
Body	9	1201	1.16	0.25 to 2.08	92.9	1.30	
Tail	9	1201	1.20	0.30 to 2.11	93	1.28	
By MRI technique							
Two-/three-echo Dixon	11	1894	1.08	0.28 to 1.88	89.3	1.11	0.85
Multi-echo Dixon	18	2052	1.16	0.70 to 1.63	90	0.74	
By world location							
Asian	17	1802	1.36	0.75 to 1.97	89.7	1.20	0.08
Non-Asian	12	2144	0.77	0.38 to 1.16	83.9	0.24	
By sample size							
*N* < 100	15	867	1.21	0.62 to 1.79	79	0.81	0.68
*N* ≥ 100	14	3059	1.05	0.48 to 1.62	93.6	0.88	
By risk of bias							
Low	12	1927	1.14	0.49 to 1.79	93.1	0.94	0.94
Moderate/high	17	2019	1.11	0.59 to 1.64	86	0.77	

*Abbreviations*: SMD standardized mean difference, MRI magnetic resonance imaging

## Discussion

In this systematic review and meta-analysis of 30 observational studies encompassing 3,980 individuals, MRI-measured IPFD was substantially higher in individuals with T2DM compared with non-diabetic controls (SMD 1.13). Subgroup analyses revealed no significant differences in the magnitude of this association by world region, MRI techniques, pancreatic region, sample size, and risk of bias. Restricting the analysis to average non-obese populations did not attenuate the effect (SMD 1.27). Despite significant heterogeneity, multiple sensitivity analyses confirmed the robustness of the observed association. In univariable meta-regressions, SMD BMI was significantly associated with increased IPFD in T2DM. Multivariable models suggested that SMD BMI may act as an independent contributor to IPFD changes, whereas SMD LFC and SMD age did not significantly modify the association. Notably, even after adjustment for all these study-level variables, a considerable residual SMD IPFD persisted (intercept = 0.797, *p* = 0.010, q = 0.018).

Our findings are consistent with the growing body of evidence highlighting the role of IPFD in T2DM pathophysiology. The PANDORA (PANcreatic Disease Originating from intRa-pancreatic fAt) hypothesis postulates a causative role of high IPFD in the development of T2DM (as well as major exocrine pancreatic diseases) [62]. This hypothesis is strongly supported by earlier longitudinal evidence [7–9, 63, 64], including evidence from lean individuals [65]. The robust association observed between T2DM and increased IPFD aligns with this hypothesis and is further supported by experimental and human data linking IPFD to β-cell dysfunction and insulin resistance, primarily mediated via lipotoxicity [5, 11, 48, 66]. Orthogonal evidence also shows that individuals with high genetic predisposition to insulin resistance have significantly higher IPFD [67]. Besides, the observed absolute increase in IPFD of 3.86% in individuals with T2DM is substantial relative to previously proposed normal thresholds [68, 69]. 

Our analyses demonstrated that the increase in IPFD among individuals with T2DM (SMD 1.13) persisted after accounting for LFC, age, and BMI. Notably, average non-obese populations with T2DM still exhibited a substantial effect (SMD 1.27) compared with controls. This finding aligns with a 2025 whole-organ quantitative histological analysis of adult donor pancreases, which reported higher adipocyte infiltration in both intra- and interlobular pancreatic compartments in individuals with T2DM versus non-diabetic controls [70]. The independence from obesity suggests that IPFD is not merely a bystander of general adiposity, but may arise through alternative mechanisms of fat accumulation in the pancreas, including lipid droplets within acinar cells and islets, adipose replacement following acinar cell apoptosis, and acinar-to-adipocyte transdifferentiation [1]. The observed gradient of IPFD from controls to T2DM reflects a continuum, reinforcing the notion that IPFD could serve as an early marker of metabolic dysfunction along the trajectory of T2DM (independent of the obesity status).

This study has several important strengths. Building on previous individual studies of MRI-measured IPFD in T2DM [9], this study is the first meta-analysis to quantify the magnitude of the association between T2DM and IPFD using MRI exclusively. Our meta-analysis includes a large sample of MRI-based IPFD measurements from diverse populations across Asia, Europe, and North America. By applying rigorous inclusion criteria centered on MRI-based measurements performed by researchers (studies using AI-based measurements were ineligible [26]) techniques and excluding MRS studies, we ensured greater consistency and accuracy in the IPFD measurements analyzed. Heterogeneity was comprehensively evaluated using multiple approaches, including subgroup analyses, univariable and multivariable meta-regressions, and advanced sensitivity analyses such as GOSH/DBSCAN clustering. Furthermore, the use of SMDs allowed harmonization of IPFD metrics across different MRI protocols—including magnet strength, acquisition parameters, ROI placement, and post-processing algorithms—enhancing comparability of pooled estimates. Finally, we examined the influence of differences in BMI, LFC, and age on excess IPFD in T2DM using multivariable meta-regression, providing a more nuanced understanding of their contributions to the observed heterogeneity.

However, the present meta-analysis has several limitations, many of which stem from the nature of the eligible studies. First, between-study heterogeneity remained considerable despite extensive exploratory analyses, likely reflecting differences in population characteristics and study design. Advanced structural heterogeneity analyses suggested that this heterogeneity was structural rather than driven by specific studies, and that the pooled estimates remained robust. Second, meta-regression analyses were conducted at the study-level, which may not accurately capture individual-level relationships, underscoring the need for future research using individual participant data. Third, the observational design of the included studies precludes causal inference regarding the relationship between IPFD and T2DM. Fourth, important clinical variables (e.g., glycemic control, duration of T2DM, and use of background medications) were inconsistently reported, limiting the ability to adjust for potential confounders and possibly contributing to residual heterogeneity. Fifth, most included studies were conducted within clinical or hospital-based settings, which may restrict the generalizability of the findings. Last, several included studies had small sample sizes per group, which may have contributed to variability in effect size estimates and between-study heterogeneity.

From a clinical perspective, our results support excessive IPFD as a robust metabolic feature associated with T2DM. In light of the PANDORA hypothesis [62], this pattern suggests that IPFD assessment may contribute to refined cardiometabolic phenotyping and potentially inform future risk stratification strategies [4]. Importantly, the utility of routine IPFD assessment remains uncertain, and our findings should not be interpreted as supporting implementation in clinical practice at present. Such a paradigm shift would require evidence on cost-effectiveness and added value over existing cardiometabolic markers. In addition, the observed differences in IPFD between T2DM and non-diabetic controls highlight an understudied therapeutic dimension [71]. Interventions such as glucose-lowering therapies, bariatric surgery, and lifestyle modification have been suggested to reduce IPFD, though the evidence remains limited [19, 20, 24, 71, 72]. These observations provide a biologically plausible rationale bridging the link between IPFD reduction and improvements in lipotoxicity, β-cell function, and insulin responsiveness [24, 73]. While causality cannot be inferred, these considerations support the interpretation that mitigating IPFD may contribute, at least in part, to the reversibility of T2DM observed in some intervention studies [74, 75]. 

Several important questions for future investigation emerge from this work. Prospective longitudinal studies are needed to elucidate the temporality of IPFD during the natural history of T2DM, ideally using serial MRI assessments in conjunction with cardiometabolic outcomes. Incorporating IPFD as an imaging endpoint in clinical trials of metabolic diseases [76, 77] would also help clarify its relevance as a therapeutic target. In parallel, further research is needed to better characterize the biological pathways linking IPFD to systemic metabolic dysfunction. Standardization of MRI protocols for IPFD quantification also remains an important methodological priority [18, 26]. Understanding IPFD as part of multiorgan ectopic fat burden may improve prediction of T2DM complications and support more refined patient stratification in precision diabetes frameworks [78, 79]. 

## Conclusion

This systematic review, meta-analysis, and meta-regression provides a comprehensive synthesis of the association between T2DM and MRI-measured IPFD, quantifying the magnitude of IPFD differences and examining the contribution of BMI, LFC, and age. The findings suggest that higher IPFD may represent a distinct metabolic feature of T2DM, independent of obesity and hepatic steatosis. Recognizing the structural pancreatic changes associated with IPFD may help reframe T2DM not only as a disease of insulin resistance and β-cell dysfunction, but also as a condition characterized by an easily and objectively measurable morphological substrate in the pancreas—an insight with important implications for prevention, risk stratification, and therapeutic targeting in this population.

## Key References

- Petrov MS, Taylor R. Intra-pancreatic fat deposition: bringing hidden fat to the fore. Nat Rev Gastroenterol Hepatol. 2022;19(3):153–168. 
  - This landmark review provides a comprehensive discussion of the role of IPFD in health and disease. It also offers insights into the epidemiology, quantification, and lability of IPFD.
- Dong X, Zhu Q, Yuan C, et al. Associations of Intrapancreatic Fat Deposition With Incident Diseases of the Exocrine and Endocrine Pancreas: A UK Biobank Prospective Cohort Study. American Journal of Gastroenterology. 2024;119(6):1158–1166.
  - Using UK Biobank data, this study shows longitudinal evidence on the association between high IPFD and the development of major exocrine and endocrine pancreatic diseases. It provides clinical evidence for positioning IPFD as a relevant therapeutic target to reduce the burden of pancreatic diseases.
- Chan TT, Tse YK, Lui RNS, et al. Fatty pancreas is independently associated with subsequent diabetes mellitus development: a 10-year prospective cohort study. Clin Gastroenterol Hepatol. 2022;20(9):2014–2022.e4.
  - This prospective cohort study provides some of the first longitudinal evidence of an independent association between high IPFD and the development of diabetes mellitus.
- Zhang J, Liu Y, Petrov MS. Validation of magnetic resonance imaging for quantification of intrapancreatic fat deposition using phantom and histologic comparators: a systematic review and meta-analysis. Eur Radiol. Published online March 23, 2026. 10.1007/s00330-026-12475-x.
  - This systematic review and meta-analysis demonstrates the high comparability of chemical shift-encoded MRI with both phantom models and histologic measurements for IPFD quantification.
- Agon HC, Shen Y, Petrov MS. Effects of glucose-lowering medications on intrapancreatic fat deposition: a systematic review and meta-analysis of randomized controlled trials. Obes Rev. 2026;27(6). 10.1111/obr.70087.
  - This systematic review and meta-analysis demonstrates the reduction of IPFD following glucose-lowering medications. As a field-wide review, it calls for future research to investigate the effects of pipeline and approved drugs for metabolic diseases on IPFD.
- Petrov MS. Fatty change of the pancreas: the Pandora's box of pancreatology. Lancet Gastroenterol Hepatol. 2023;8(7):671–682.
  - In this personal view, the PANDORA hypothesis is introduced as a comprehensive framework that positions IPFD as a central morphological and pathobiological determinant across major pancreatic diseases.

## Supplementary Information

Below is the link to the electronic supplementary material.

Supplementary Material 1

## Data Availability

Available from the corresponding author on reasonable request.

## Abbreviations

IPFD: intrapancreatic fat deposition
T2DM: type 2 diabetes mellitus
MRS: magnetic resonance spectroscopy
MRI: magnetic resonance imaging
PDFF: proton density fat fraction
PRISMA: Preferred Reporting Items for Systematic reviews and Meta-Analyses
MOOSE: Meta-analysis Of Observational Studies in Epidemiology
PROSPERO: International Prospective Register of Systematic Reviews
BMI: body mass index
ROI: region of interest
LFC: liver fat content
CI: confidence interval
REML: restricted maximum likelihood
SMD: standardized mean difference
FDR: false discovery rate
GOSH: Graphic Display of Study Heterogeneity
DBSCAN: density-based clustering
PANDORA: PANcreatic Disease Originating from intRa-pancreatic fAt
