# Supplementary material for: Burden of Intrapancreatic Fat Deposition in Type 2 Diabetes and the Role of Obesity: A Systematic Review and Meta-Analysis
Source: Curr Obes Rep. 2026 May 21;15(1):40. doi: 10.1007/s13679-026-00718-3 (PMC13190432; doi:10.1007/s13679-026-00718-3)

**SUPPLEMENTARY MATERIAL**

**Burden of Intrapancreatic Fat Deposition in Type 2 Diabetes and the Role of Obesity: A Systematic Review and Meta-analysis**

**Summary of contents**

SUPPLEMENTARY METHODS2

SUPPLEMENTARY TABLES3

SUPPLEMENTARY REFERENCES15

SUPPLEMENTARY FIGURES17

**SUPPLEMENTARY METHODS**

***Search strategy***

We searched the (A) PubMed and (B) Embase databases (from inception to November 15, 2025) using the following search strategy:

1. **PubMed**

( ("fatty pancreas"[tiab] OR "pancreatic steatosis"[tiab] OR "pancreatic fat"[tiab] OR "intrapancreatic fat"[tiab] OR "pancreatic lipomatosis"[tiab] OR "nonalcoholic fatty pancreas disease"[tiab] OR "non-alcoholic fatty pancreas disease"[tiab] OR "NAFPD"[tiab] OR (pancrea*[tiab] AND (fat*[tiab] OR steato*[tiab] OR ectopic[tiab] OR lipomatosis[tiab] OR hyperecho*[tiab]))) ) AND ( "Diabetes Mellitus, Type 2"[Mesh] OR "diabetes mellitus"[tiab] OR "type 2 diabetes"[tiab] OR "type II diabetes"[tiab] OR "T2D"[tiab] OR "T2DM"[tiab] OR diabetic[tiab] ) AND ( "Humans"[Mesh] ) NOT ( "Animals"[Mesh] NOT "Humans"[Mesh] )

1. **Embase**

( (pancrea* OR intrapancreatic) NEAR/3 (ectopic OR fatty OR fat OR steato* OR NAFPD OR lipomatosis OR hyperecho*) ):ti,ab,kw AND ( 'diabetes mellitus'/exp OR 'type 2 diabetes mellitus'/exp OR diabetes:ti,ab,kw OR diabetic:ti,ab,kw OR 'type 2 diabetes':ti,ab,kw OR 'type II diabetes':ti,ab,kw OR T2D:ti,ab,kw OR T2DM:ti,ab,kw ) AND 'human'/de NOT ('animal'/de NOT 'human'/de)

**SUPPLEMENTARY TABLES**

**Table S1.** MRI characteristics of included studies.

| **First author (year)** | **MRI technique** | **Field strength** | **Vendor** | **Acquisition parameters** | **Pancreatic region measured** | **Lead image analyst** | **Measurement scale** |
| --- | --- | --- | --- | --- | --- | --- | --- |
| Ma et al.^1^ (2014) | Six-echo IDEAL-Quant | 3.0T | GE (Signa HDx) | TR 14 ms; TE 1.59 / 3.17 / 4.76 / 7.59 / 9.70 / 11.8 ms; BW 125 kHz; ETL 6; flip 5°; NEX 0.72; matrix 256×160; 20 slices; thickness 6 mm; 24-s breath-hold. | Head (1 ROI) | Single observer | PDFF (%)^a^ |
| Kühn et al.^2^ (2015) | Three-echo chemical shift–encoded MRI | 1.5T | Siemens (Magnetom Avanto) | TR/TE1/TE2/TE3 = 11/2.4/4.8/9.6 ms; flip 10°; BW 1065 Hz/pixel; FOV 410×308 mm; matrix 224×168×64; 3D GRE; parallel imaging (GRAPPA 1.8); single 19-s breath-hold; axial acquisition | Head, body, tail (3 ROIs); **(only whole-pancreas IPFD available for T2DM; regional values not reported**) | - | PDFF (%)^a^ |
| Idilman et al.^3^ (2015) | Six-echo IDEAL-IQ | 1.5T | GE (HDxt) | TR 12.9 ms; TE range 1.6–9.8 ms (6 echoes); flip 5°; FOV 35–40 cm; matrix 224×160; BW 125 kHz; slice 5 mm; single 3D slab (44–56 slices); breath-hold <25 s; 2D parallel imaging (acceleration 2) | Head, body, tail (1 ROI per region; ~1 cm²; avoiding ducts, vessels, collecting systems) | Radiologist, blinded to biopsy results | PDFF (%)^a^ |
| Macauley et al.^4^ (2015) | Three-echo Dixon | 3.0T | Philips (Achieva) | TR 50 ms; TE = 3.45/4.60/5.75 ms; flip 5°; BW 435 Hz/px; 12 slices; 5-mm slice thickness; four 17-s breath-holds | Three central slices of homogeneous pancreatic parenchyma (manual polygon ROIs; avoiding ducts, vessels, visceral fat) | Trained analysts (two independent observers; blinded) | Fat fraction (%)^b^ |
| Chai et al.^5^ (2016) | Two-echo Dixon | 3.0T | GE (Signa Excite) | TR 224 ms; TE (IP/OP) 2.4 / 5.8 ms; flip 80°; echo train length 17; NEX 2; slice 5 mm; matrix 288×192; breath-hold; supine position | Head, body, tail (3 ROIs ~155 mm²; placed centrally; avoiding vessels and adjacent fat) | - | Fat fraction (%)^b^ |
| Heber et al.^6^ (2017) | Six-echo Dixon | 3.0T | Siemens (Magnetom Skyra) | TR 8.90 ms; TE1–TE6 = 1.23 / 2.46 / 3.69 / 4.92 / 6.15 / 7.38 ms; flip 4°; slice 4 mm; FOV read 420 mm / phase 78.1%; 15-s breath-hold; full correction for T1, T2*, noise bias, fat spectrum; automated PDFF output archived to PACS. | Head, body, tail (3 ROIs ~100 mm²; multiple slices; artifacts excluded) | Readers blinded to diabetes status; inter- and intra-reader ICC reported (0.95 and 0.80) | PDFF (%)^a^ |
| Lu et al.^7^ (2019) | Three-echo Dixon | - | - | TR 9.25 ms; TE 2.45/3.67/7.35 ms; flip 9°; slice 5 mm; NEX 1; FOV 400×400 mm; BW 270 Hz/pixel; breath-hold 17–21 s; supine position | Head, body, tail (3 ROIs → mean) | - | Fat fraction (%)^b^ |
| Wang et al.^8^ (2019) | Six-echo IDEAL-IQ | 1.5T | GE Healthcare (Brivo MR355) | TR = 15.6 ms; 6 echoes per TR (TE1 = 1.2–1.5 ms; ΔTE = 1.23 ms × 6 echoes); flip angle = 8°; slice thickness = 10 mm; standard IDEAL-IQ reconstruction (water, fat, R2*, PDFF maps) | Head, body, tail (3 ROIs ≈10–15 mm²); ROIs fully surrounded by pancreatic parenchyma, avoiding ducts, vessels, and collecting systems | Radiologist with >6 years of abdominal MRI experience; blinded to clinical/biochemical data | PDFF (%)^a^ |
| Tirkes et al.^9^ (2019) | Two-echo Dixon | 3.0T | Siemens (Magnetom Verio) | TR 5.45 ms; TE1 2.45 ms; TE2 3.675 ms; flip angle 9°; slice thickness 4 mm; axial breath-hold acquisition; T1-weighted GRE | ROIs placed in axial slices | Two image analysts (ROI placement) + abdominal radiologist (16 years experience, blinded, verified segmentation) | Fat fraction (%)^b^ |
| Nadarajah et al.^10^ (2020) | Two-echo Dixon | 1.5T or 3.0T | Siemens (Magnetom Avanto, Harmony, Verio) | 3T: TR 5.45 ms; TE 2.45/3.67 ms; flip 10°; BW 320 Hz/pixel; FOV 450×450 mm; matrix 256×128 / 1.5T: TR 7.53 ms; TE 4.76/2.38 ms; flip 10°; BW 300 Hz/pixel; FOV 400×587 mm; matrix 256×256 / Fasting 6 h; upper abdomen coverage | Head, body, tail (3 ROIs; avoiding ducts, vessels, artifacts, visceral fat) | Primary reviewer (15 years experience), blinded; subset checked by second reviewer (4 years experience) | Fat fraction (%)^b^ |
| Sarma et al.^11^ (2020) | Six-echo Dixon | 3.0T | Siemens (Prisma 3T) | TE1 = 1.23 ms with ΔTE = 1.23 ms (equidistant); TR = 8.85 ms; flip angle = 5°; slice thickness = 3 mm; matrix = 320×240; FOV ≈ 360×270 mm²; bandwidth = 1080 Hz/px; GRAPPA factor 2; 38–52 axial slices covering T12–L5; enhanced water–fat separation + PCA-based denoising; multi-peak fat model and T2* correction included in signal model | Head, body, tail - pancreas segmented with region-growing algorithm in SliceOmatic. Whole-pancreas mean PDFF computed | Single trained observer supervised by an experienced radiologist (blinded) | PDFF (%)^a^ |
| Li et al.^12^ (2021) | Three-echo mDixon | 3.0T | Philips (Ingenia II) | Imaging time 14 s; TR 5.5 ms; TE1 0.92 ms; flip 3°; matrix 152×113; slice 6 mm; gap 3 mm; FOV 380×285×330 mm; supine position; upper abdomen acquisition; T1WI | Whole pancreas; half-pancreas (hPFF); plus ROIs (50 mm²) in head, body, tail; avoidance of vessels and peripancreatic fat | Two observers; double-blind; independent measurements | Fat fraction (%)^b^ |
| Zheng et al.^13^ (2022) | Two-echo Dixon | 1.5T | Siemens (MAGNETOM Aera) | TR 6.46 ms; TE1/TE2 2.39/4.77 ms; FOV 380×380 mm; in-plane resolution 1.3×1.3 mm; slice 3 mm; acquisition 15 s; breath-hold | Head, neck, body, tail (4 ROIs ~1.5 cm²; mean of 4) | - | Fat fraction (%)^b^ |
| Waddell et al.^14^ (2022) | Ten-echo gradient-recalled echo (GRE) acquisition | 1.5T | Siemens (Aera 1.5T) | TE1 = 2.38 ms; ΔTE = 2.38 ms; magnitude-based multipoint water–fat separation algorithm | Head, body, tail - single 10-mm ROI placed in each region, avoiding ducts and vessels | Manual ROI placement | PDFF (%)^a^ |
| Wen et al.^15^ (2022) | Six-echo IDEAL-IQ | 3.0T | GE Healthcare (Discovery 750W) | TR 6.4 ms; TE 6 ms; FOV 50×50 cm; slice thickness 6.0 mm; matrix 256×160; bandwidth 111.1 kHz; flip angle 5°; NEX 0.5; breath-hold 15–20 s | Head, body, tail - each measured three times with circular ROI (100 mm²), avoiding vessels and adjacent adipose tissue | Radiologist (3 years experience), blinded; repeated measurements at 30-day interval; averaged | PDFF (%)^a^ |
| Yu et al.^16^ (2023) | Six-echo IDEAL-IQ | 3.0T | GE (Discovery MR750) | TR 15.6 ms; TE1 1.2–1.5 ms (ΔTE 1.23 ms); flip 8°; slice 10 mm; breath-hold; fat-suppressed T2WI + in/opposed-phase T1WI also acquired | Head, body, tail (ROIs ~10–15 mm²; mean of 3) | Radiologist (>5 years experience), blinded | PDFF (%)^a^ |
| Yi et al.^17^ (2023) | Six-echo qDixon-WIP | 3.0T | Siemens (Prisma) | TE 1.26 / 2.60 / 3.94 / 5.28 / 6.62 / 7.96 ms; TR 9.25 ms; slice 3.5 mm; matrix 160×120; BW 1040 Hz/pixel; FOV 380×313.5 mm; breath-hold ~18 s; abdominal axial acquisition | Head, body, tail (3 ROIs ~0.1–0.2 cm²; mean of 3) | Two radiologists (>5 years experience), independent measurements | PDFF (%)^a^ |
| Cao et al.^18^ (2023) | Six-echo Dixon ( | 3.0T | - | TE1–TE6 = 1.05 / 2.46 / 3.69 / 4.92 / 6.15 / 7.38 ms; TR = 9.0 ms; flip angle 4°; FOV 420×420 mm; slice thickness 3.5 mm; axial multi-echo Dixon breath-hold acquisition | Whole pancreas - manual segmentation of all slices on PDFF maps, avoiding ducts, vessels, adjacent visceral fat | Radiologists (not specified), blinded | PDFF (%)^a^ |
| Yasokawa et al.^19^ (2023) | Six-echo 3D mDIXON Quant | 3.0T | Philips (Ingenia 3T CX Quasar Dual) | TR per mDixon Quant default; flip angle 4°; slice thickness 3–4 mm; PDFF parametric maps automatically generated on the scanner | Head, body, tail - largest possible circular/oval ROI in each region; avoiding ducts, vessels, retroperitoneal fat, artifacts; mean of 3 regions = whole-pancreas PDFF | Two fellowship-trained abdominal radiologists (blinded), each performing two independent measurements | PDFF (%)^a^ |
| Ting et al.^20^ (2023) | Three-echo Dixon | - | - | TR 9.25 ms; TE 2.45/3.67/7.35 ms; flip 9°; slice 5 mm; NEX 1; FOV 400×400 mm; BW 270 Hz/pixel | Head, body, tail (3 ROIs) | - | Fat fraction (%)^b^ |
| Wang et al.^21^ (2023) | 2-echo Dixon | 3.0T | Siemens (Magnetom Prisma) | TE1 = 1.23 ms; TE2 = 2.46 ms; TR = 3.97 ms; flip angle = 9°; bandwidth = 1040 Hz/pixel; slice thickness = 3.0 mm; breath-hold at end expiration; overnight fasting (10–12 h) | Whole pancreas (3D coverage; ROI placement across entire gland; strict exclusion of vessels, ducts, organ edges, lesions, artifacts) | Primary radiologist (same for all processing); second radiologist (>4 years experience), blinded | Fat fraction (%)^b^ |
| An et al.^22^ (2024) | Multi-echo IDEAL-IQ (1.5T / 3T) and mDixon Quant (3T) | 1.5T or 3.0T | GE (Signa HDxt) and Philips (Ingenia CX) | IDEAL-IQ 1.5T: TR 13.4 ms; TE 4.8 ms; FOV 36×36 cm; matrix 256×160; NEX 1; slice 10 mm; flip 5° / IDEAL-IQ 3T: TR 6.9 ms; TE 3.0 ms; FOV 36×36 cm; matrix 256×160; NEX 1; slice 10 mm; flip 3° / mDixon Quant 3T: TR 6 ms; TE 1.05 ms; FOV 37×30 cm; matrix 176×130; slice 5 mm; flip 3° Breath-hold acquisition; multiple echoes; reconstruction with water/fat/R2*/PDFF maps | Whole pancreas (3D semi-automatic segmentation on IntelliSpace Portal; fully parenchymal) | Two radiologists; blinded | PDFF (%)^a^ |
| Diamond et al.^23^ (2024) | Multi-echo Dixon | 1.5T or 3.0T | Siemens (Prisma 3T, Skyra 3T, Aera 1.5T) and GE (Signa Voyager 1.5T) | PDFF generation via CoverScan algorithm | Whole pancreas parenchyma | MRI technologists/radiographers; blinded | PDFF (%)^a^ |
| Qu et al.^24^ (2025) | Multi-echo Dixon | 1.5T | Siemens (Magnetom Avanto) | Single breath-hold; phased-array 16-channel coil; fasting ≥10 h; fitting algorithm for fat quantification | Head, body, tail (3 ROIs; manual; avoiding vessels, duct, visceral fat, artifacts) | Single experienced radiologist, blinded | PDFF (%)^a^ |
| Elsayed et al.^25^ (2025) | Six-echo Dixon | 3.0T | Philips (Achieva) | Automatic R2* and fat-fraction maps; breath-hold 17 s; scan time 5–10 min; fasting 4–6 h; T2WI, T2*WI and Dixon acquisition | Head, body, tail (3 ROIs; avoiding duct, vessels, extra-pancreatic fat; mean of 3) | - | Fat fraction (%)^b^ |
| Yuan et al.^26^ (2025) | Six-echo mDixon Quant | 3.0T | Philips (Ingenia 3.0T) | TE1–TE6 = 11.04 / 11.84 / 12.64 / 13.24 / 14.04 / 14.84 ms; TR = 6.0 ms; flip angle 4°; FOV 200×400×105 mm; slice thickness 6 mm; axial acquisition. | Head, body, tail (3 ROIs) | Two radiologists (3 and 6 years’ experience), blinded; measurements performed using Philips IPS workstation | PDFF (%)^a^ |
| Akhan et al.^27^ (2025) | 2-echo Dixon | 1.5T | Siemens (Magnetom Aera 1.5T) | TR = 6.9 ms; TE1 = 2.3 ms; TE2 = 4.8 ms; flip angle = 10°; FOV = 40 cm; bandwidth = 475 Hz/pixel; matrix = 240 × 320; acquisition time = 12 s; breath-hold 3D Dixon acquisition generating in-phase, out-of-phase, water-only, and fat-only images | Head, body, tail (3 circular ROIs 100 mm² each); ROIs carefully avoiding ducts, vessels, and non-parenchymal fat | Two abdominal radiologists (4 and 12 years of experience), blinded to clinical data | Fat fraction (%)^b^ |
| Nie et al.^28^ (2025) | Multi-echo Dixon | 3.0T | Philips (Ingenia Elition X) | breath-hold & fasting state | Head, body, tail (3 ROIs 100 mm² each); avoided vessels and ducts | Radiologist with 8 years of experience | PDFF (%)^a^ |
| Nielsen et al.^29^ (2025) | Multi-echo Dixon | 3.0T | GE Healthcare (Signa Premier 3T) | TR 5.7 ms; TE (minimum full); slice thickness 5 mm; FOV 44 cm; matrix 160×160; flip angle 3°; breath-hold acquisition | Whole pancreas | - | PDFF (%)^a^ |
| Zhu et al.^30^ (2025) | Multi-echo Dixon | 3.0T | Philips (Ingenia 3.0T) | Transverse-axis mDixon T1-weighted sequence; | Head, body, tail (3 ROIs), avoiding vessels and surrounding adipose tissue | Two experienced abdominal radiologists, blinded to clinical information | PDFF (%)^a^ |

^a^ PDFF (%) = proton density fat fraction, including studies explicitly reporting PDFF or using validated quantitative multi-echo Dixon-based fat-fraction mapping methods intended to estimate PDFF.

^b^ Fat fraction (%) = fat fraction estimated by two-/three-echo Dixon, or other non-PDFF approaches.

**Table S2.** Characteristics of populations in included studies.

| **First author (year)** | **T2DM diagnosis** | **T2DM** | | | | | **No T2DM** | | | | |
| --- | --- | --- | --- | --- | --- | --- | --- | --- | --- | --- | --- |
|  |  | **Sample size, n** | **Women, %** | **BMI, kg/m^2^** | **Age, years** | **HbA1c, % or mmol/L** | **Sample size, n** | **Women, %** | **BMI, kg/m^2^** | **Age, years** | **HbA1c. % or mmol/L)** |
| Ma et al.^1^ (2014) | Abnormal OGTT/ elevated fasting glucose | 24 | 45.8 | - | Mean 44.88 {range 27-61} | - | 10 | 40.0 | - | Mean 45.5 {range 29-60} | - |
| Kühn et al.^2^ (2015) | Abnormal OGTT/ elevated fasting glucose | 70 | 41.4 | Median 31.0 [IQR 27.8-33.5] | Median 60 [IQR 54-68] | - | 740 | 60.3 | Median 25.4 [IQR 23.1-28.4] | Median 45 [IQR 36-55] | - |
| Idilman et al.^3^ (2015) | - | 5 | - | - | - | - | 36 | - | - | - | - |
| Macauley et al.^4^ (2015) | Abnormal OGTT/ elevated fasting glucose | 41 | - | Mean 30.3 (SEM 0.5) | Mean 61.8 (SEM 1.0) | Mean 6.4 (SEM: 0.1) | 14 | - | Mean 29.6 (SEM 1.0) | Mean 59.0 (SEM 2.2) | - |
| Chai et al.^5^ (2016) | Elevated fasting glucose | 70 | 22.9 | Mean 26.89 (SD 4.56) | Mean 43.99 (SD 1.32) | - | 30 | 46.7 | Mean 25.45 (SD 2.8) | Mean 42.65 (SD 9.7) | - |
| Heber et al.^6^ (2017) | Abnormal OGTT/ elevated fasting glucose | 53 | 24.5 | Median 30.4 [IQR 27-33] | Median 63 [IQR 58-69] | - | 237 | 48.5 | Median 26.2 [IQR 23.7-28.9] | Median 53 [IQR 47-62] | - |
| Lu et al.^7^ (2019) | 2010 Chinese T2DM Guidelines | 78 | 39.7 | Mean 25.66 (SD 4.17) | Mean 58.19 (SD 9.62) | - | 35 | 51.4 | Mean 23.00 (SD 2.60) | Mean 58.33 (SD 7.09) | - |
| Wang et al.^8^ (2019) | ADA 2013 criteria | 15 | 53.3 | - | Mean 51.0 (SD 8.0) | - | 16 | 50.0 | - | Mean 50.0 (SD 10.7) | - |
| Tirkes et al.^9^ (2019) | Medical records | 16 | 75.0 | Median 57 {range 28–76} | Median 31 {range 21–44} | - | 102 | 73.5 | Median 28 {range 17–47} | Median 55 {range 19–85} | - |
| Nadarajah et al.^10^ (2020) | Medical records | 45 | 53.3 | Mean 31.1 (SD 7.3) | Mean 59.9 (SD 13.1) | Mean 7.44 (SD 1.78) | 150 | 55.3 | Mean 30.2 (SD 7) | Mean 52.5 (SD 13.2) | - |
| Sarma et al.^11^ (2020) | Medical records | 14 | 57.1 | Mean 26.3 (SD 3.7) | Mean 57.5 (SD 7.2) | Mean 8.2 (SD 2.1) | 13 | 53.8 | Mean 25.7 (SD 1.8) | Mean 58.8 (SD 7.7) | - |
| Li et al.^12^ (2021) | Medical records | 7 | - | - | - | - | 53 | - | - | - | - |
| Zheng et al.^13^ (2022) | WHO 1999 criteria | 118 | 39.0 | - | Mean 57.07 (SD 13.41) | - | 39 | 74.4 | - | Mean 52.33 (SD 12.03) | - |
| Waddell et al.^14^ (2022) | Self-reported | 131 | 32.1 | Median 29.4 [IQR 27-33.9] | Median 56 [IQR 52-63] | Median 42.9 mmol/L (IQR 38.6-51.3) | 135 | 26.7 | Median 29.7 [IQR 26.8-33.5] | Median 57 [IQR 53-63] | Median 36.1 mmol/L (IQR 32.9-38.3) |
| Wen et al.^15^ (2022) | Abnormal OGTT/ elevated fasting glucose | 16 | - | - | - | - | 18 | 0.0 | - | - | - |
| Yu et al.^16^ (2023) | ADA 2021 criteria | 28 | 46.4 | Median 39.1 [IQR 35.3–43.3] | Mean 29.0 (SD 8.3) | Median 8.7 (IQR 7.1–10.6) | 54 | 48.1 | Median 36.4 [IQR 31.7–41.0] | Mean 31.3 (SD 8.9) | Median 5.5 (IQR 5.1–5.7) |
| Yi et al.^17^ (2023) | - | 47 | 36.2 | Mean 23.04 (SD 2.826) | Mean 51.32 (SD 10.604) | - | 48 | 39.6 | Mean 23.68 (SD 2.327) | Mean 51.28 (SD 8.907) | - |
| Cao et al.^18^ (2023) | ADA 2022 criteria | 40 | 32.5 | Median 32.70 [IQR 30.30-36.13] | Median 33 [IQR 28-40] | Median 7.90 (IQR 6.60-10.98) | 52 | 42.3 | Median 32.95 [IQR 29.60-35.73] | Median 30 [IQR 25-35] | Median 5.30 (IQR 5.07-5.50) |
| Yasokawa et al.^19^ (2023) | ADA 2018 criteria | 23 | - | - | Mean 68.2 (SD 7.7) | - | 32 | - | - | Mean 65.2 (SD 8) | - |
| Ting et al.^20^ (2023) | - | 70 | 42.9 | Mean 25.78 (SD 4.02) | Mean 58.46 (SD 9.82) | - | 30 | 50.0 | Mean 22.89 (SD 2.69) | Mean 57.96 (SD 7.01) | - |
| Wang et al.^21^ (2023) | ADA 2018 criteria | 80 | 25.0 | Mean 29.74 (SD 4.81) | Mean 42.89 (SD 10.93) | Median 10.30 (IQR 8.90–11.33) | 20 | 35.0 | Mean 21.37 (SD 1.85) | Mean 42.55 (SD 13.14) | Median 5.15 (IQR 5.10–5.30) |
| An et al.^22^ (2024) | Elevated fasting glucose/on glucose-lowering medications | 68 | 41.2 | Mean 25.49 (SD 2.54) | Mean 63.81 (SD 12.35) | - | 273 | 57.1 | Mean 24.41 (SD 3.09) | Median 57 [IQR 49-64] | - |
| Diamond et al.^23^ (2024) | - | 112 | - | - | - |  | 91 | - | - | - |  |
| Qu et al.^24^ (2025) | WHO 1999 criteria | 40 | 27.5 | Median 26.83 [IQR 25.57-29.96] | Mean 38.53 (SD 12.31) | Median 9.9 (IQR 7.4-11.7) | 40 | 12.5 | Median 27.51 [IQR 25.84-30.06] | Mean 36.08 (SD 11.44) | Median 5.6 (IQR 5.4-5.8) |
| Elsayed et al.^25^ (2025) | ADA 2023 criteria | 15 | 33.3 | Mean 29.65 (SD 5.07) | Mean 51.60 (SD 12.12) | Mean 8.16 (SD 1.83) | 15 | 40.0 | Mean 26.69 (SD 5.72) | Mean 28.93 (SD 3.04) | Mean 5.37 (SD 0.17) |
| Yuan et al.^26^ (2025) | 2020 Chinese T2DM Guidelines | 80 | 23.8 | Mean 28.72 (SD 0.86) | Mean 47.39 (SD 8.18) | Mean 9.15 | 38 | 42.1 | Mean 23.50 (SD 0.52) | Mean 46 (SD 11) | - |
| Akhan et al.^27^ (2025) | ADA criteria | 55 | - | Mean 32.81 (SD 4.88) | Mean 51.64 (SD 8.72) | Mean 8.45 (SD 2.27) | 31 | - | Mean 25.4 (SD 4.36) | Mean 45.13 (SD 8.31) | Mean 5.34 (SD 0.3) |
| Nie et al.^28^ (2025) | Abnormal OGTT/ elevated fasting glucose | 46 | 50.0 | Median 29.8 [IQR 28.0-32.5] | Median 68.5 [IQR 65.8-71.8] | - | 50 | 48.0 | Median 29.2 [IQR 28.0, 31.3] | Median 68.2 [IQR 65.3-72.2] | - |
| Nielsen et al.^29^ (2025) | ADA 2025 criteria | 12 | 83.3 | Mean 22.3 (SD 5.5) | Mean 46.8 (SD 11.5) | Median 45 (IQR 44–55) | 11 | 54.5 | 24.5 (4.6) | Mean 36.0 (SD 13.0) | Median 36 (IQR 32–42) |
| Zhu et al.^30^ (2025) | WHO 1999 criteria | 74 | 51.3 | - | Mean 58.37 (SD 9.71) | - | 74 | 52.7 | - | Mean 59.56 (SD 10.08) | - |

**Abbreviations:** T2DM, type 2 diabetes mellitus; IQR, interquartile range; SD, standard deviation; SEM, standard error of the mean.

**Table S3.** Risk of bias in included studies.

Cross-sectional design

| **First author (year)** | **Selection** | | | | **Comparability** | **Outcome** | | **Risk of bias (NOS)** |
| --- | --- | --- | --- | --- | --- | --- | --- | --- |
|  | **Bias due to representativeness of the sample - Domain scoring: 0 (High); 1 (Low)** | **Bias due to sample size - Domain scoring: 0 (High); 1 (Low)** | **Bias due to non-respondents - Domain scoring: 0 (High); 1 (Low)** | **Bias due to ascertainment of the exposure (risk factor) - Domain scoring: 0 (High); 1 (Moderate); 2 (Low)** | **Bias due to comparability - Domain scoring: 0 (High); 1 (Moderate); 2 (Low)** | **Bias due to assessment of outcome - Domain scoring: 0 (High); 1 (Moderate); 2 (Low)** | **Bias due to statistical test - Domain scoring: 0 (High); 1 (Low)** |  |
| Ma et al.^1^ (2014) | 0 | 0 | 0 | 2 | 0 | 1 | 1 | High (4) |
| Kühn et al.^2^ (2015) | 1 | 1 | 0 | 2 | 2 | 1 | 1 | Moderate (8) |
| Idilman et al.^3^ (2015) | 0 | 0 | 1 | 2 | 0 | 1 | 1 | High (5) |
| Macauley et al.^4^ (2015) | 1 | 0 | 1 | 2 | 1 | 2 | 1 | Moderate (8) |
| Chai et al.^5^ (2016) | 1 | 1 | 1 | 1 | 0 | 1 | 1 | High (6) |
| Lu et al.^7^ (2019) | 0 | 0 | 0 | 2 | 2 | 2 | 1 | Moderate (7) |
| Wang et al.^8^ (2019) | 1 | 0 | 1 | 2 | 1 | 2 | 1 | Moderate (8) |
| Tirkes et al.^9^ (2019) | 0 | 1 | 1 | 2 | 2 | 2 | 1 | Low (9) |
| Nadarajah et al.^10^ (2020) | 0 | 1 | 1 | 2 | 2 | 2 | 1 | Low (9) |
| Sarma et al.^11^ (2020) | 0 | 0 | 1 | 2 | 2 | 1 | 1 | Moderate (7) |
| Li et al.^12^ (2021) | 0 | 0 | 1 | 2 | 0 | 1 | 1 | High (5) |
| Zheng et al.^13^ (2022) | 1 | 1 | 1 | 1 | 1 | 2 | 1 | Moderate (8) |
| Waddell et al.^14^ (2022) | 1 | 1 | 1 | 2 | 2 | 1 | 1 | Low (9) |
| Wen et al.^15^ (2022) | 1 | 0 | 1 | 2 | 1 | 2 | 1 | Moderate (8) |
| Yu et al.^16^ (2023) | 1 | 1 | 0 | 2 | 2 | 2 | 1 | Low (9) |
| Yi et al.^17^ (2023) | 1 | 1 | 1 | 2 | 2 | 2 | 1 | Low (10) |
| Cao et al.^18^ (2023) | 1 | 1 | 1 | 2 | 1 | 2 | 1 | Low (9) |
| Yasokawa et al.^19^ (2023) | 0 | 1 | 1 | 2 | 0 | 2 | 1 | Moderate (7) |
| Ting et al.^20^ (2023) | 0 | 0 | 0 | 2 | 1 | 2 | 1 | High (6) |
| Wang et al.^21^ (2023) | 1 | 1 | 0 | 1 | 1 | 2 | 1 | Moderate (7) |
| An et al.^22^ (2024) | 1 | 1 | 1 | 2 | 2 | 2 | 1 | Low (10) |
| Diamond et al.^23^ (2024) | 0 | 1 | 1 | 2 | 0 | 2 | 1 | Moderate (7) |
| Qu et al.^24^ (2025) | 1 | 0 | 0 | 2 | 2 | 2 | 1 | Moderate (8) |
| Yuan et al.^26^ (2025) | 1 | 1 | 1 | 2 | 2 | 2 | 1 | Low (10) |
| Akhan et al.^27^ (2025) | 1 | 1 | 1 | 2 | 1 | 2 | 1 | Low (9) |
| Nie et al.^28^ (2025) | 0 | 1 | 1 | 2 | 2 | 2 | 1 | Low (9) |
| Zhu et al.^30^ (2025) | 1 | 1 | 1 | 2 | 1 | 2 | 1 | Low (9) |

Case-control design

| **First author (year)** | **Selection** | | | | **Comparability** | **Exposure** | | | **Risk of bias (NOS)** |
| --- | --- | --- | --- | --- | --- | --- | --- | --- | --- |
|  | **Bias due to definition of the cases - Domain scoring: 0 (High); 1 (Low)** | **Bias due to representativeness of the cases - Domain scoring: 0 (High); 1 (Low)** | **Bias due to selection of controls - Domain scoring: 0 (High); 1 (Low)** | **Bias due to definition of controls - Domain scoring: 0 (High); 1 (Low)** | **Bias due to comparability - Domain scoring: 0 (High); 1 (Moderate); 2 (Low)** | **Bias due to ascertainment of exposure - Domain scoring: 0 (High); 1 (Low)** | **Bias due to method of ascertainment for cases and controls - Domain scoring: 0 (High); 1 (Low)** | **Bias due to non-response rate - Domain scoring: 0 (High); 1 (Low)** |  |
| Heber et al.^6^ (2017) | 1 | 1 | 1 | 1 | 2 | 1 | 1 | 0 | Low (8) |
| Elsayed et al.^25^ (2025) | 1 | 0 | 0 | 1 | 1 | 1 | 1 | 1 | Moderate (6) |
| Nielsen et al.^29^ (2025) | 1 | 1 | 1 | 1 | 1 | 1 | 1 | 0 | Moderate (7) |

**SUPPLEMENTARY REFERENCES**

1. Ma J, Song Z, Yan F. Detection of hepatic and pancreatic fat infiltration in type II diabetes mellitus patients with IDEAL-Quant using 3.0T MR: comparison with single-voxel proton spectroscopy. *Chin Med J (Engl)*. 2014;127(20):3548-3552.

2. Kühn JP, Berthold F, Mayerle J, et al. Pancreatic steatosis demonstrated at MR imaging in the general population: clinical relevance. *Radiology*. 2015;276(1):129-136. doi:10.1148/RADIOL.15140446

3. Idilman IS, Tuzun A, Savas B, et al. Quantification of liver, pancreas, kidney, and vertebral body MRI-PDFF in non-alcoholic fatty liver disease. *Abdom Imaging*. 2015;40(6):1512-1519. doi:10.1007/s00261-015-0385-0

4. Macauley M, Percival K, Thelwall PE, Hollingsworth KG, Taylor R. Altered volume, morphology and composition of the pancreas in type 2 diabetes. *PLoS One*. 2015;10(5):e0126825. doi:10.1371/journal.pone.0126825

5. Chai J, Liu P, Jin E, et al. MRI chemical shift imaging of the fat content of the pancreas and liver of patients with type 2 diabetes mellitus. *Exp Ther Med*. 2016;11(2):476-480. doi:10.3892/etm.2015.2925

6. Heber SD, Hetterich H, Lorbeer R, et al. Pancreatic fat content by magnetic resonance imaging in subjects with prediabetes, diabetes, and controls from a general population without cardiovascular disease. *PLoS One*. 2017;12(5):e0177154. doi:10.1371/journal.pone.0177154

7. Lu T, Wang Y, Dou T, Xue B, Tan Y, Yang J. Pancreatic fat content is associated with β-cell function and insulin resistance in Chinese type 2 diabetes subjects. *Endocr J*. 2019;66(3):265-270. doi:10.1507/endocrj.EJ18-0436

8. Wang M, Luo Y, Cai H, et al. Prediction of type 2 diabetes mellitus using noninvasive MRI quantitation of visceral abdominal adiposity tissue volume. *Quant Imaging Med Surg*. 2019;9(6):1076-1086. doi:10.21037/qims.2019.06.01

9. Tirkes T, Jeon CY, Li L, et al. Association of pancreatic steatosis with chronic pancreatitis, obesity, and type 2 diabetes mellitus. *Pancreas*. 2019;48(3):420-426. doi:10.1097/MPA.0000000000001252

10. Nadarajah C, Fananapazir G, Cui E, et al. Association of pancreatic fat content with type II diabetes mellitus. *Clin Radiol*. 2020;75(1):51-56. doi:10.1016/j.crad.2019.05.027

11. Sarma MK, Saucedo A, Darwin CH, et al. Noninvasive assessment of abdominal adipose tissues and quantification of hepatic and pancreatic fat fractions in type 2 diabetes mellitus. *Magn Reson Imaging*. 2020;72:95-102. doi:10.1016/j.mri.2020.07.001

12. Li X, Yang Q, Ye H, Li S, Wang Y, Yu W. Comparison of pancreatic fat content measured by different methods employing MR mDixon sequence. *PLoS One*. 2021;16(11):e0260001. doi:10.1371/journal.pone.0260001

13. Zheng Y, Yang S, Chen X, Lv J, Su J, Yu S. The correlation between type 2 diabetes and fat fraction in liver and pancreas: a study using MR Dixon technique. *Contrast Media Mol Imaging*. 2022;2022:7073647. doi:10.1155/2022/7073647

14. Waddell T, Bagur A, Cunha D, et al. Greater ectopic fat deposition and liver fibroinflammation and lower skeletal muscle mass in people with type 2 diabetes. *Obesity*. 2022;30(6):1231-1238. doi:10.1002/oby.23425

15. Wen Y, Chen C, Kong X, et al. Pancreatic fat infiltration, β-cell function and insulin resistance: a study of the young patients with obesity. *Diabetes Res Clin Pract*. 2022;187:109860. doi:10.1016/j.diabres.2022.109860

16. Yu X, Huang YH, Feng YZ, Cheng ZY, Cai XR. Well-controlled versus poorly controlled diabetes in patients with obesity: differences in MRI-evaluated pancreatic fat content. *Quant Imaging Med Surg*. 2023;13(6):3496-3507. doi:10.21037/qims-22-1083

17. Yi J, Xu F, Li T, et al. Quantitative study of 3T MRI qDixon-WIP applied in pancreatic fat infiltration in patients with type 2 diabetes mellitus. *Front Endocrinol (Lausanne)*. 2023;14:1140111. doi:10.3389/fendo.2023.1140111

18. Cao MJ, Wu WJ, Chen JW, et al. Quantification of ectopic fat storage in the liver and pancreas using six-point Dixon MRI and its association with insulin sensitivity and β-cell function in patients with central obesity. *Eur Radiol*. 2023;33(12):9213-9222. doi:10.1007/s00330-023-09856-x

19. Yasokawa K, Kanki A, Nakamura H, et al. Changes in pancreatic exocrine function, fat and fibrosis in diabetes mellitus: analysis using MR imaging. *Br J Radiol*. 2023;96:20210515. doi:10.1259/bjr.20210515

20. Ting L, Liyun W, Zheng W, Cao Z. Pancreatic fat content plays an important role in the development of type 2 diabetes mellitus similar to that of liver fat content. *Acta Endocrinol (Buchar)*. 2023;19(4):421-425. doi:10.4183/aeb.2023.421

21. Wang J, Cai Q, Wu X, et al. Association between intrapancreatic fat deposition and lower high-density lipoprotein cholesterol in individuals with newly diagnosed T2DM. *Int J Endocrinol*. 2023;2023:6991633. doi:10.1155/2023/6991633

22. An Q, Zhang QH, Wang Y, et al. Association between type 2 diabetes mellitus and body composition based on MRI fat fraction mapping. *Front Public Health*. 2024;12:1332346. doi:10.3389/fpubh.2024.1332346

23. Diamond C, Pansini M, Hamid A, et al. Quantitative imaging reveals steatosis and fibroinflammation in multiple organs in people with type 2 diabetes: a real-world study. *Diabetes*. 2024;73(8):1285-1299. doi:10.2337/db23-0926

24. Qu H, Zhou L, Tang D, et al. Relationship between liver fat, pancreatic fat, and new-onset type 2 diabetes mellitus in patients with metabolic dysfunction-associated fatty liver disease. *Acta Diabetol*. 2025;62(10):1725-1732. doi:10.1007/s00592-025-02501-7

25. Elsayed RA, Abo-Elhoda PM, Said NM, Sheha AS. Quantification of ectopic fat fractions in type 2 diabetes mellitus using MR-dixon technique. *Egypt J Radiol Nucl Med*. 2025;56(1):95. doi:10.1186/s43055-025-01500-6

26. Yuan B, Tao Z, Ma Y, Zhang Y, Chu Z, Ji Q. Quantitative assessment of abdominal ectopic fat deposits in patients with different glucose tolerance by using mDixon Quant MRI. *Sci Rep*. 2025;15(1):7359. doi:10.1038/s41598-025-92004-w

27. Akhan BS, Yiğit H, Aral Y, Omma T, Karaca A, Koşar PN. Advanced imaging of pancreatic steatosis with Dixon MRI: key clinical factors revealed. *Endocrinol Res Pract*. 2025;29(3):163-170. doi:10.5152/erp.2025.24572

28. Nie H, Liu M, Duan J, Liu H. Research on the relationship between ectopic fat and iron deposition in the liver and pancreas, with glucose metabolism in elderly obese patients. *Diabetes Metab Syndr Obes*. 2025; 18:2331-2341. doi:10.2147/DMSO.S518292

29. Nielsen SR, Gjela M, Stoico MP, et al. Mild pancreatic fibrosis with preserved exocrine function and increased visceral adipose tissue in m.3243A&gt;G carriers: a magnetic resonance imaging study. *Mol Genet Metab*. 2025;146(4):109282. doi:10.1016/j.ymgme.2025.109282

30. Zhu Q, Ma B, Li P, Chen W, Xu L, Qian W. Study of the correlation between pancreatic fat deposition and type 2 diabetes mellitus by quantitative MR fat analysis. *Int J Diabetes Dev Ctries*. Published online November 13, 2025. doi:10.1007/s13410-025-01569-9

**SUPPLEMENTARY** **FIGURES**

**Figure S1.** Leave-one-out sensitivity analysis.


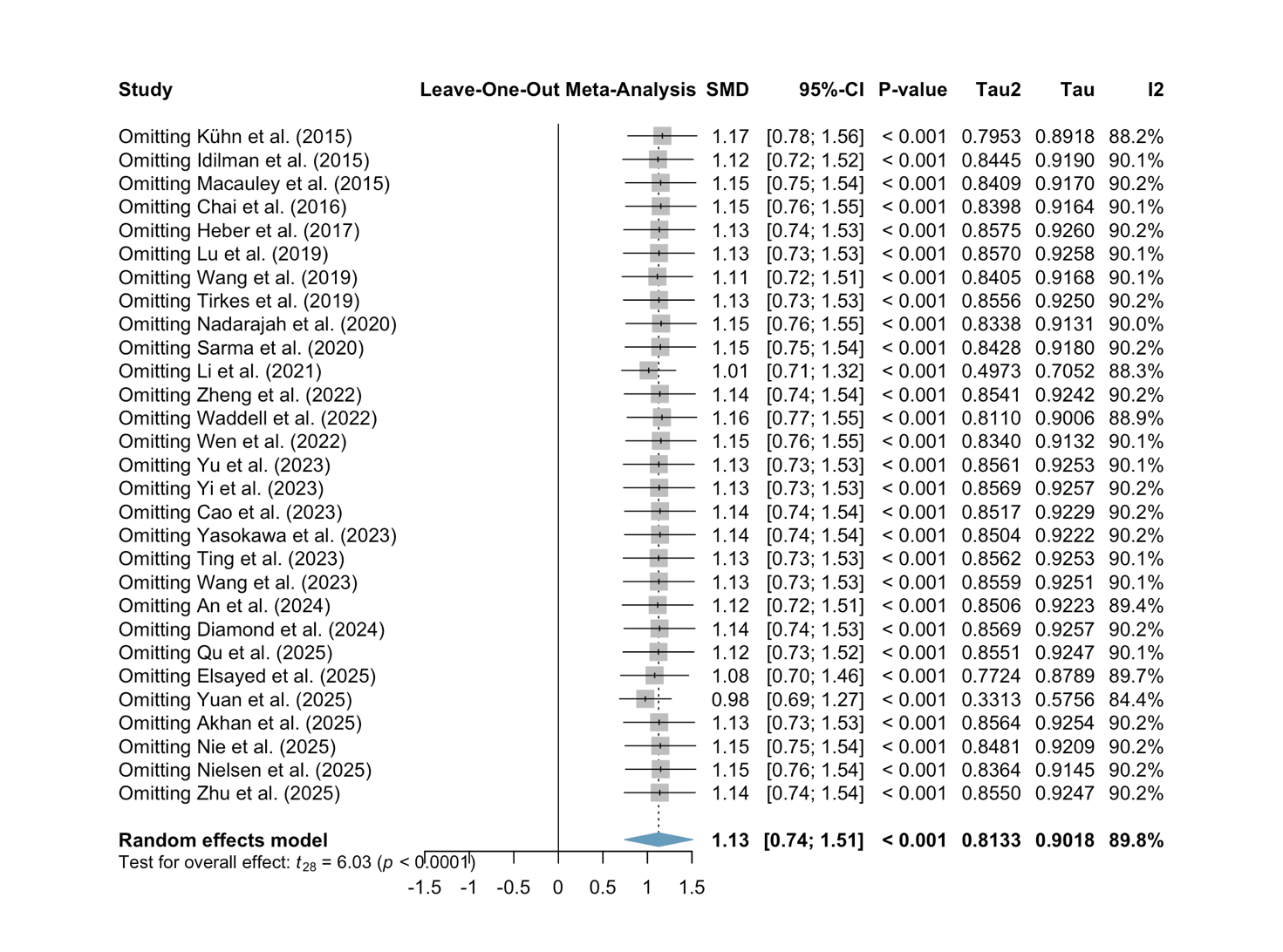


**Figure S2.** Funnel plot for the primary outcome.


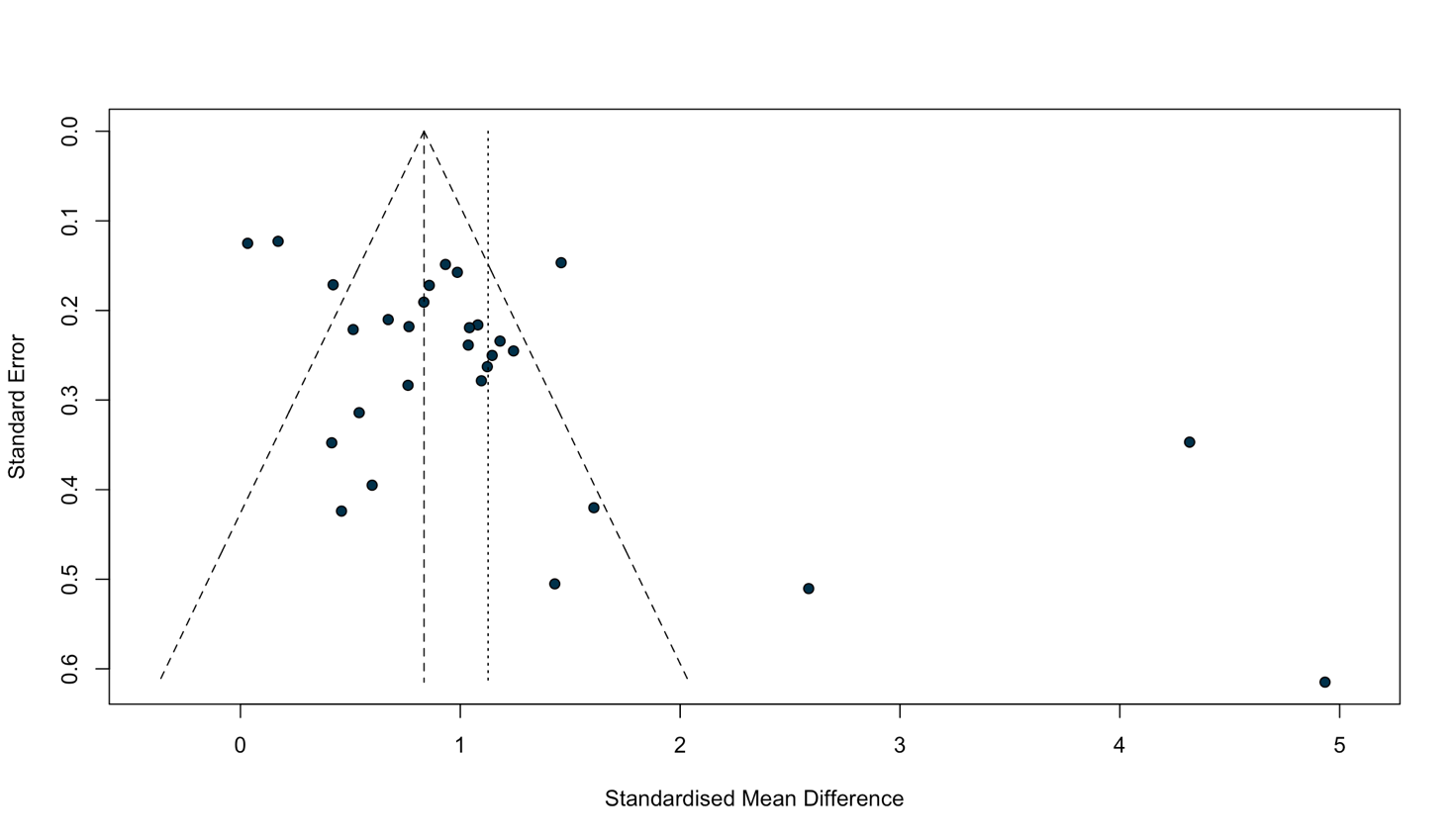

Supplement: Supplementary file 1 — Supplementary Material 1 [file 13679_2026_718_MOESM1_ESM.docx]
